# Supplementary figures and images for: Tumor-preventing activity of aspirin in multiple cancers based on bioinformatic analyses
Source: PeerJ. 2018 Sep 26;6:e5667. doi: 10.7717/peerj.5667 (PMC6163034; doi:10.7717/peerj.5667)

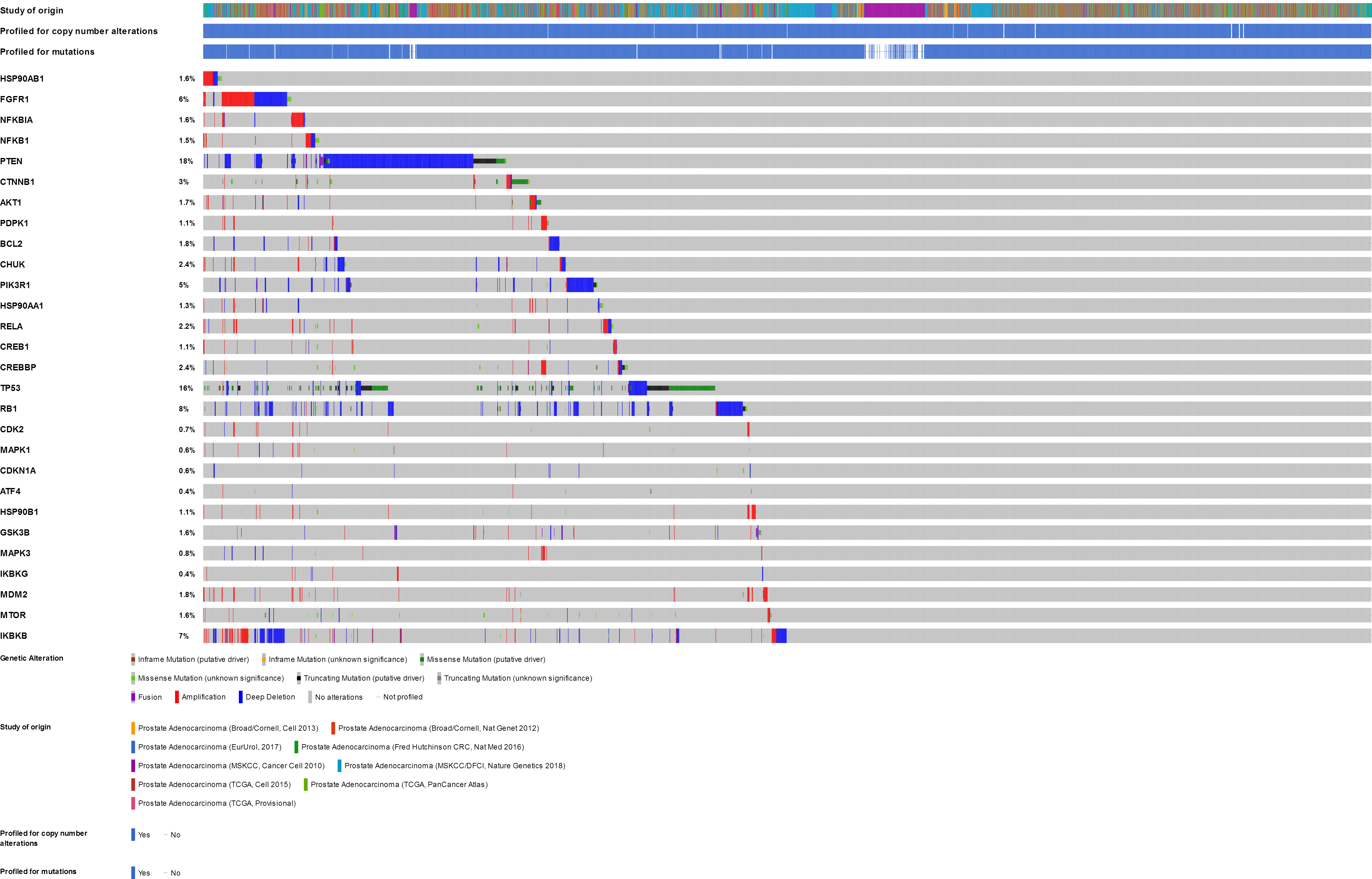

Supplement: Figure S1 [file peerj-06-5667-s001.png]

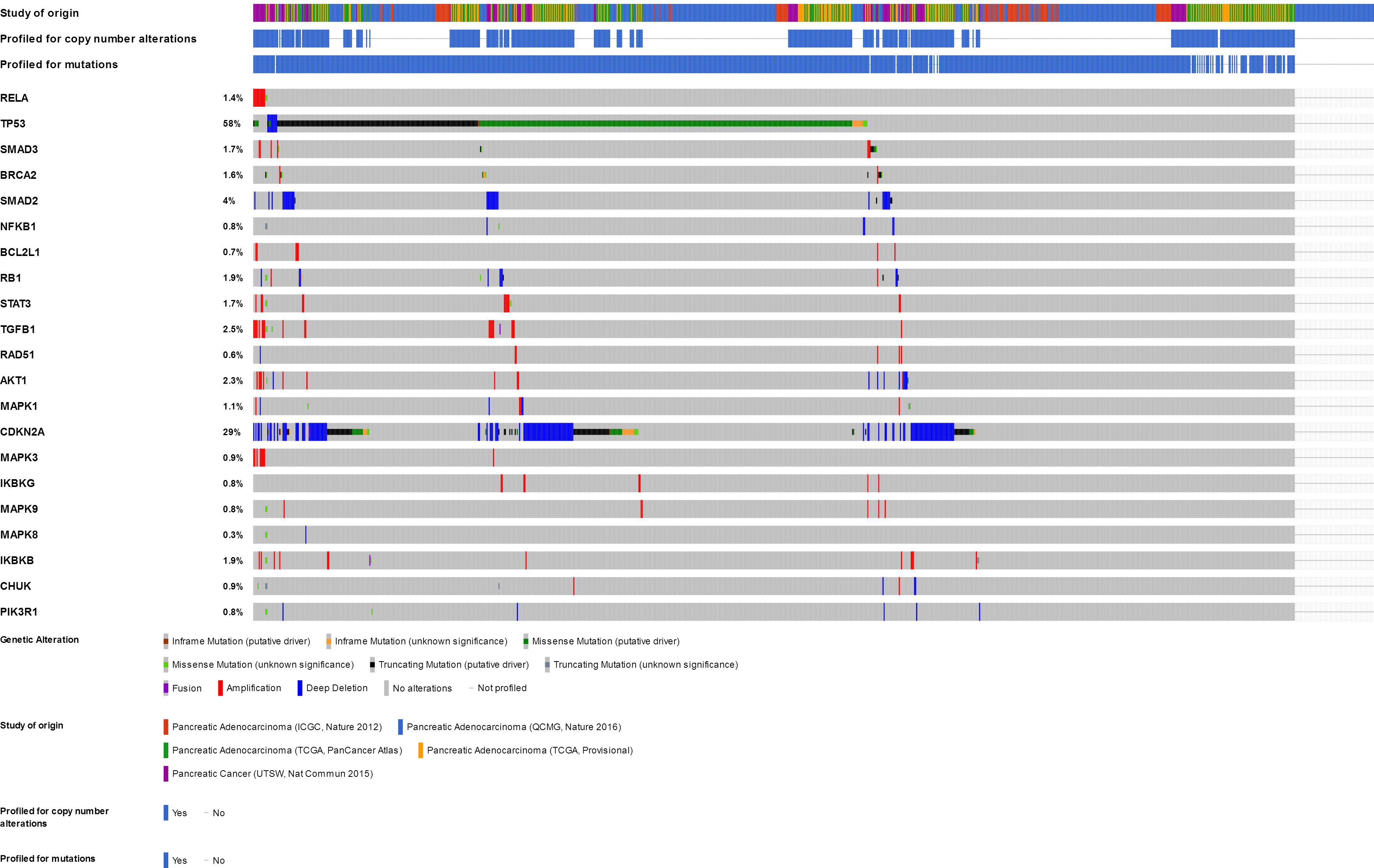

Supplement: Figure S2 [file peerj-06-5667-s002.png]

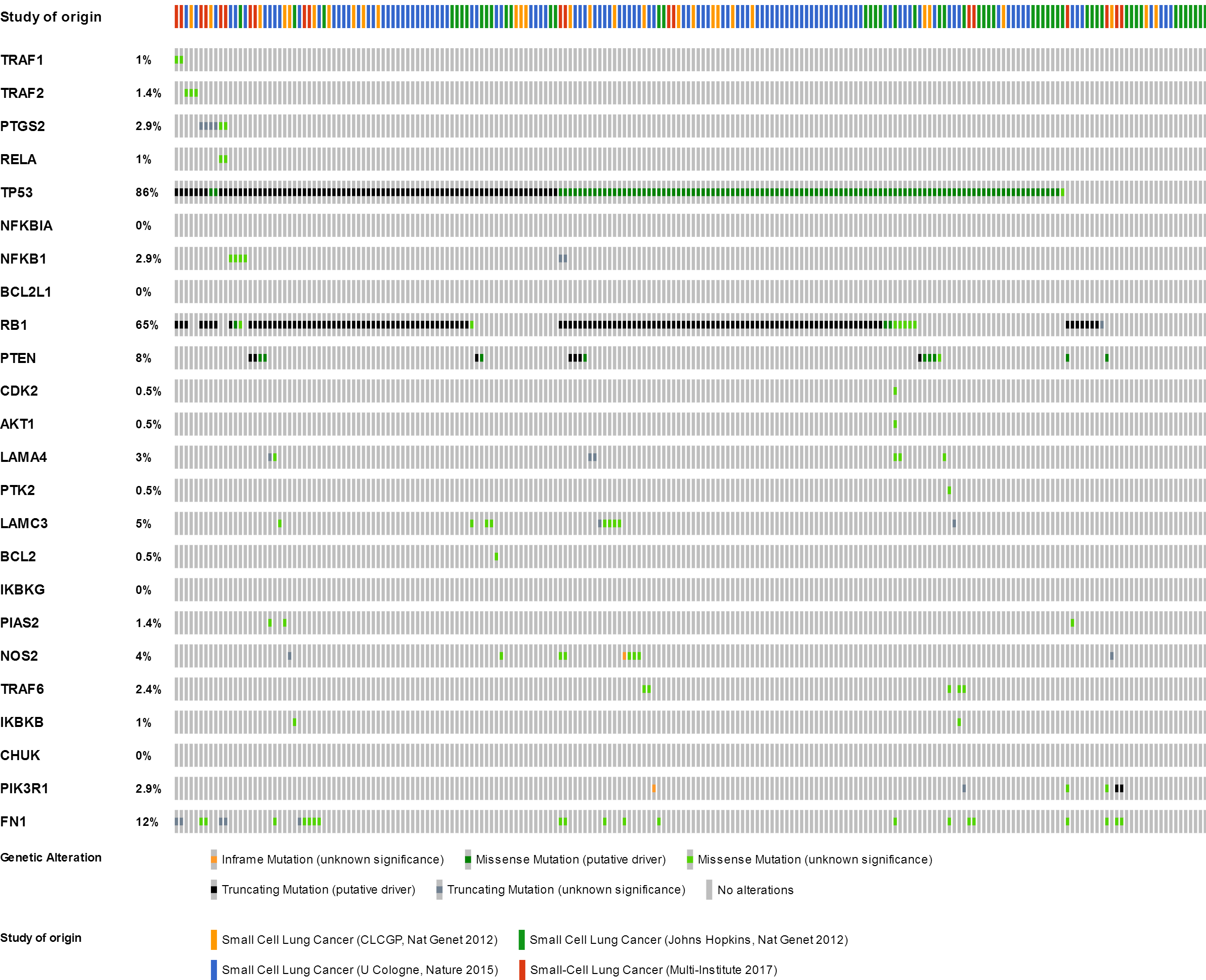

Supplement: Figure S3 [file peerj-06-5667-s003.png]

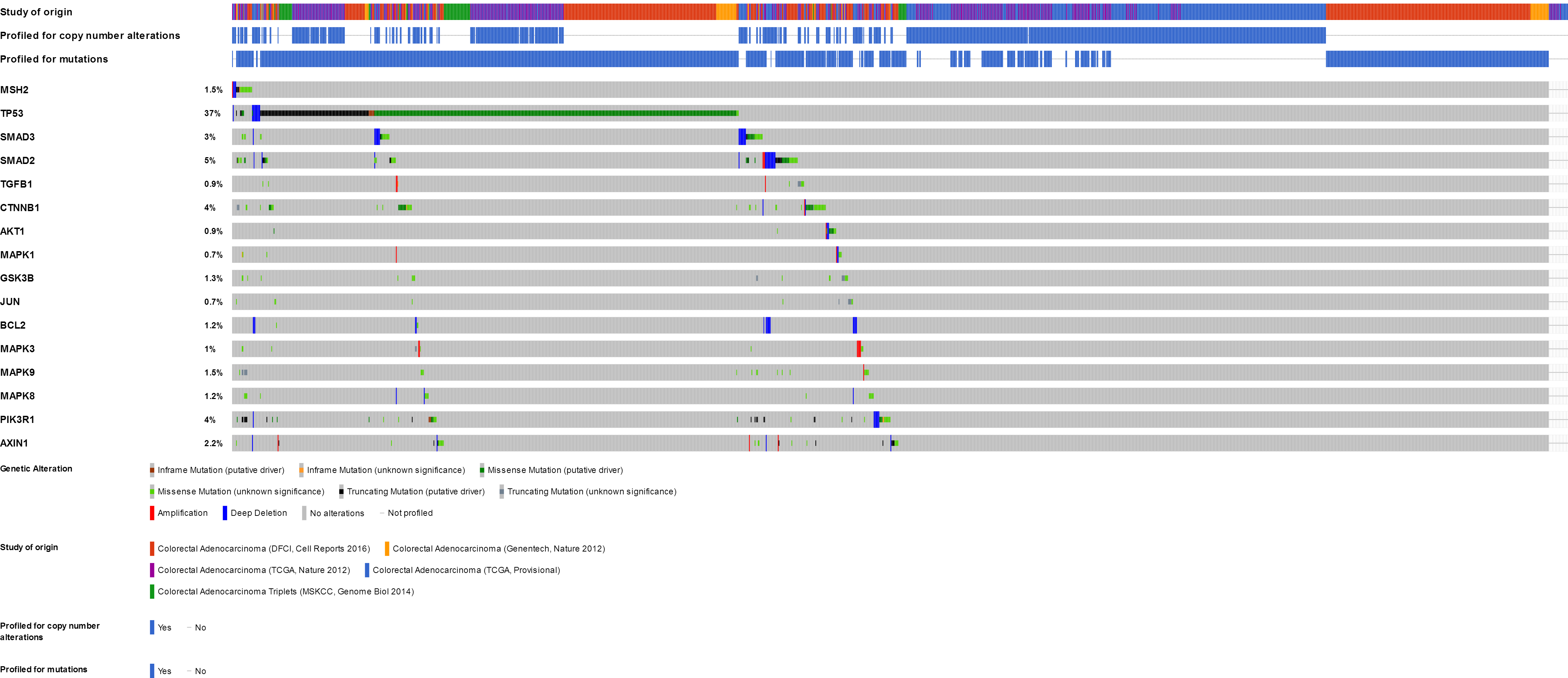

Supplement: Figure S4 [file peerj-06-5667-s004.png]

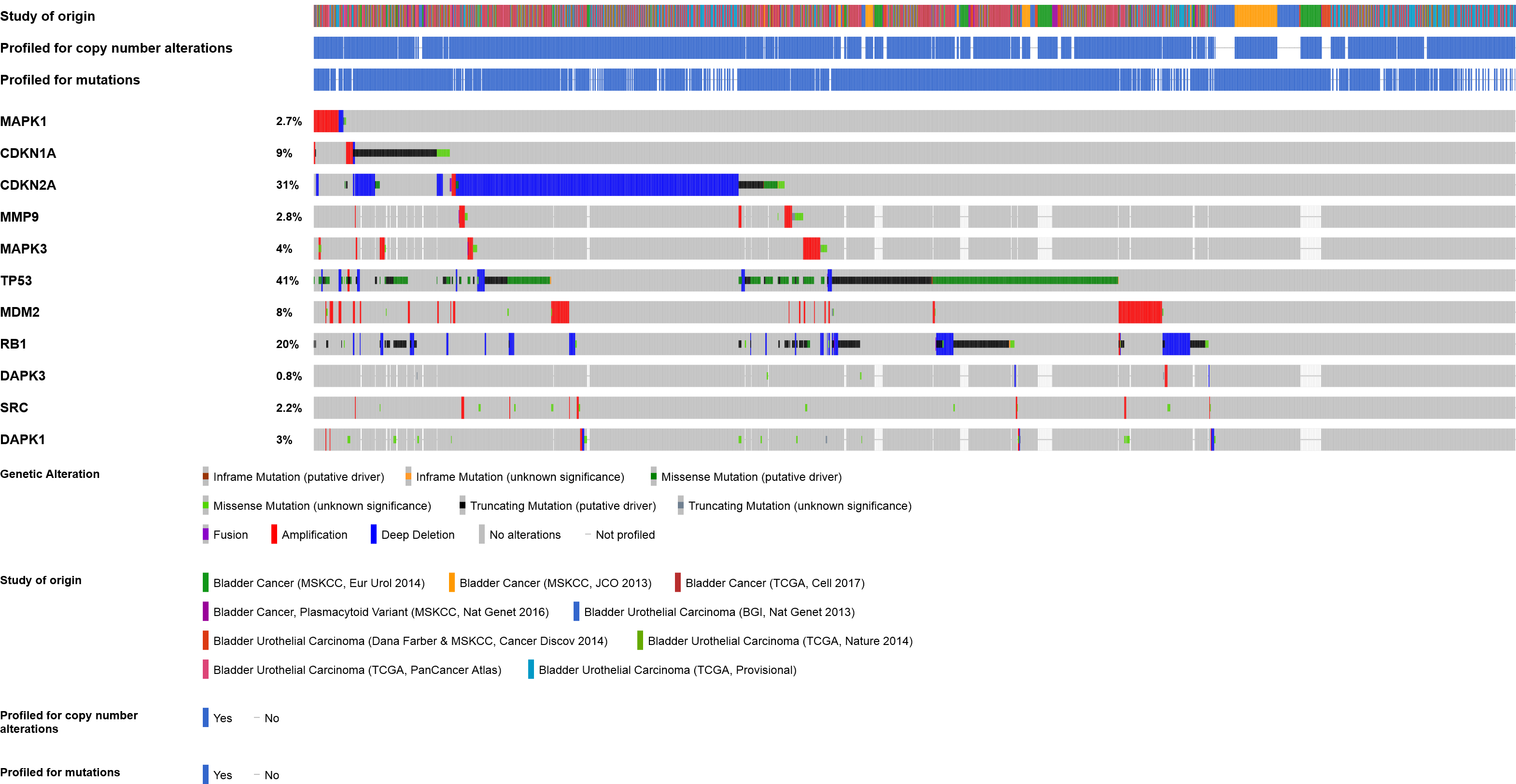

Supplement: Figure S5 [file peerj-06-5667-s005.png]

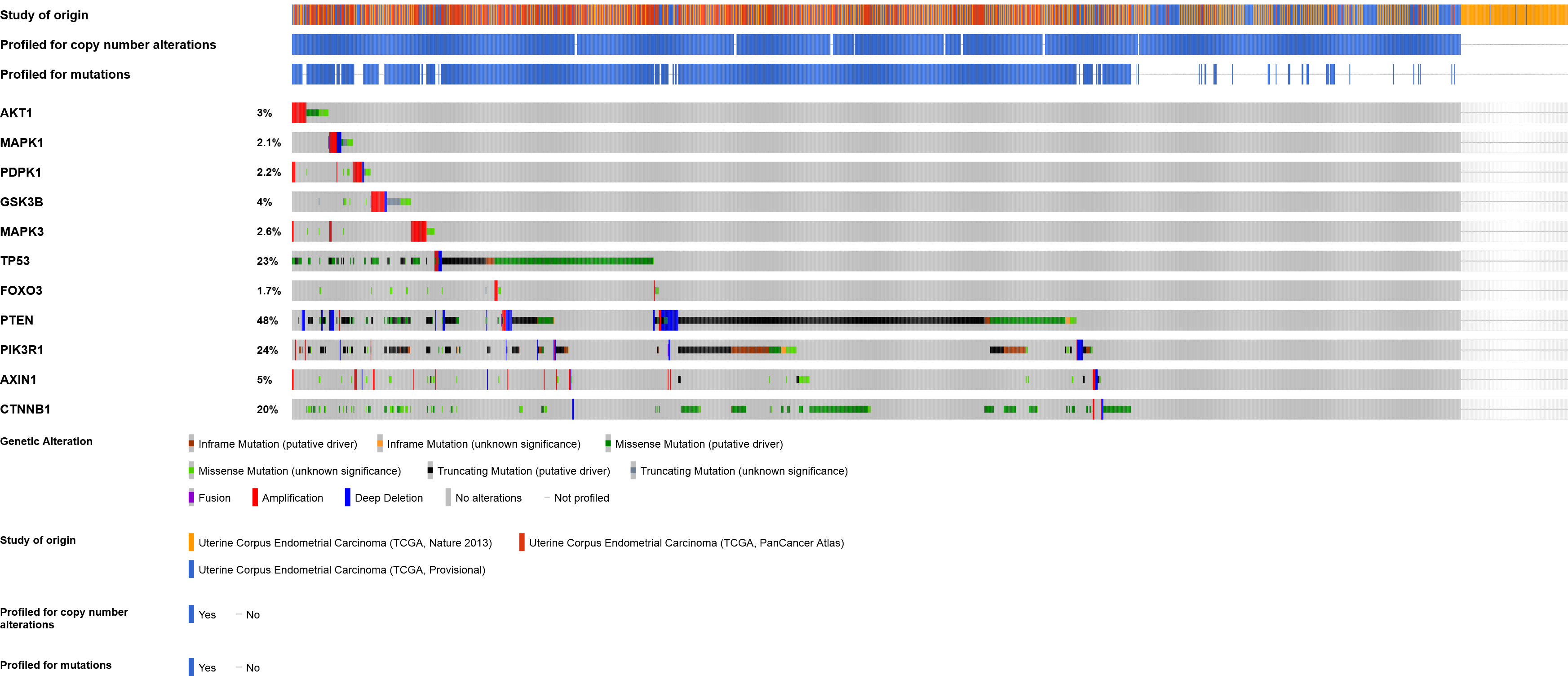

Supplement: Figure S6 [file peerj-06-5667-s006.png]

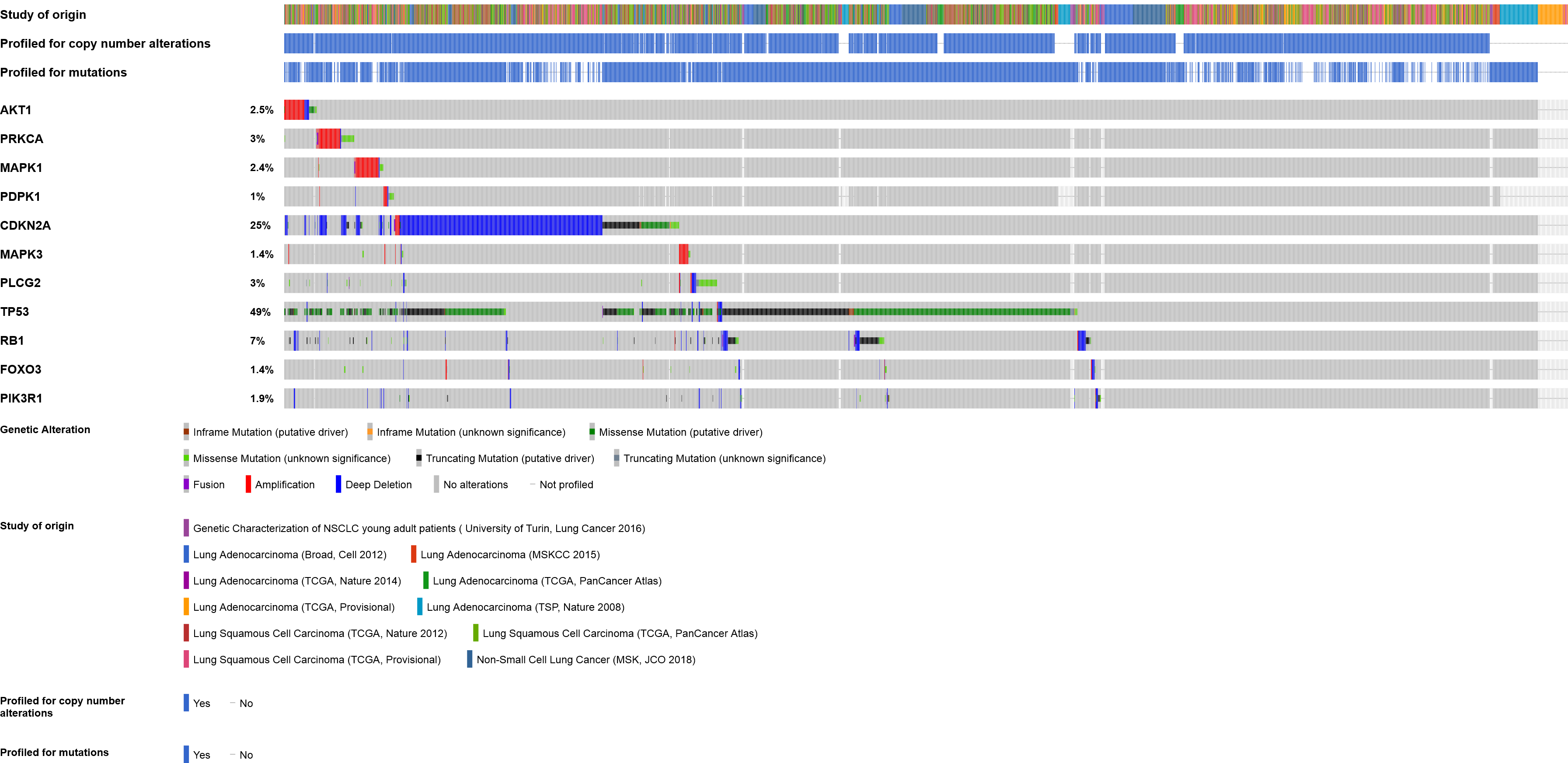

Supplement: Figure S7 [file peerj-06-5667-s007.png]

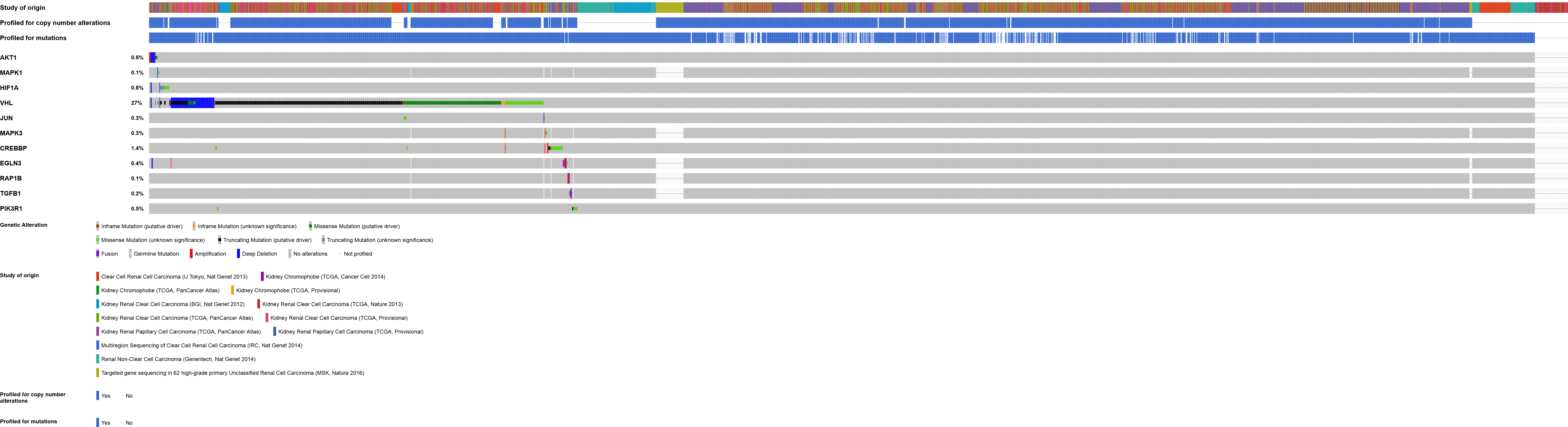

Supplement: Figure S8 [file peerj-06-5667-s008.png]
